# FRET Imaging of Diatoms Expressing a Biosilica-Localized Ribose Sensor

**DOI:** 10.1371/journal.pone.0033771

**Published:** 2012-03-21

**Authors:** Kathryn E. Marshall, Errol W. Robinson, Shawna M. Hengel, Ljiljana Paša-Tolić, Guritno Roesijadi

**Affiliations:** 1 Marine Biotechnology, Marine Sciences Laboratory, Pacific Northwest National Laboratory, Sequim, Washington, United States of America; 2 Environmental Molecular Sciences Laboratory, Pacific Northwest National Laboratory, Richland, Washington, United States of America; University of Crete, Greece

## Abstract

Future materials are envisioned to include bio-assembled, hybrid, three-dimensional nanosystems that incorporate functional proteins. Diatoms are amenable to genetic modification for localization of recombinant proteins in the biosilica cell wall. However, the full range of protein functionalities that can be accommodated by the modified porous biosilica has yet to be described. Our objective was to functionalize diatom biosilica with a reagent-less sensor dependent on ligand-binding and conformational change to drive FRET-based signaling capabilities. A fusion protein designed to confer such properties included a bacterial periplasmic ribose binding protein (R) flanked by CyPet (C) and YPet (Y), cyan and yellow fluorescent proteins that act as a FRET pair. The structure and function of the CRY recombinant chimeric protein was confirmed by expression in *E. coli* prior to transformation of the diatom *Thalassiosira pseudonana*. Mass spectrometry of the recombinant CRY showed 97% identity with the deduced amino acid sequence. CRY with and without an N-terminal Sil3 tag for biosilica localization exhibited characteristic ribose-dependent changes in FRET, with similar dissociation constants of 123.3 µM and 142.8 µM, respectively. The addition of the Sil3 tag did not alter the affinity of CRY for the ribose substrate. Subsequent transformation of *T. pseudonana* with a vector encoding *Sil3-CRY* resulted in fluorescence localization in the biosilica and changes in FRET in both living cells and isolated frustules in response to ribose. This work demonstrated that the nano-architecture of the genetically modified biosilica cell wall was able to support the functionality of the relatively complex Sil3-CyPet-RBP-YPet fusion protein with its requirement for ligand-binding and conformational change for FRET-signal generation.

## Introduction

The construction of future three-dimensional materials with multiscale architectures is expected to include bio-assembly [1]. Self-assembly through biosynthesis in living organisms may substitute for chemical synthesis of hybrid structures with functional elements immobilized in highly ordered biomineral structures like those found in nature. The biosilica cell walls of diatoms have been recognized for some time as hierarchically ordered, mesoporous, micro-to-nanoscale structures that can serve as the basis for development of advanced materials [2]. Efforts to construct silica materials inspired by an understanding of diatom biology have included 1) silica condensation from silicic acid *in vitro* with the use of silaffins or silaffin-derived peptides [3]–[7] and 2) manipulation of living cells to add functional elements by metabolic insertion [8], [9] or genetic modification of the cell wall structure [10], [11]. The latter cell-based approaches allow assembly under the ambient physical and chemical conditions inherent to diatom cell culture.

Recent advances in the development of diatom transformation systems have made it possible to construct expression vectors that can target the localization of recombinant proteins to the biosilica cell wall [10]. Green fluorescent protein (GFP) and enzymes with multimeric structure and/or have cofactor requirements have been successfully immobilized in the biosilica of *Thalassiosira pseudonana* by tagging them with the silaffin Sil3, which targets localization to the biosilica cell wall [10], [12]. Our objective was to test the ability of the diatom biosilica to serve as a scaffold for complex chimeric fusion proteins requiring large-scale motions associated with ligand-dependent conformational changes in order to function.

To accomplish this, we built a ribose sensor that uses a signaling system based on changes in Förster Resonance Energy Transfer (FRET) for localization in the diatom biosilica. The sensor construct included the bacterial periplasmic ribose binding protein (RBP; [13]) flanked by the fluorescent FRET pair CyPet and YPet [14]–[16], creating the CyPet-RBP-YPet (CRY) sensor cassette, which requires ribose binding and a conformational change by RBP to drive changes in FRET. Insertion of the silaffin *Sil3* sequence upstream of *CRY* targeted the chimeric protein for localization in the diatom biosilica. Here, we report the successful functionalization of *T. pseudonana* with a complex reagent-less sensor immobilized in the biosilica cell wall. This research demonstrates the potential for the diatom system to accommodate complex proteins in a three-dimensional hybrid material through bioassembly under ambient conditions.

## Results

### Construction and Characterization of the CRY Sensor

The design of the CRY recombinant sensor was based on the *FLIPrbs- F15A* construct (mutant form F15A; [13]) encoding RBP flanked by enhanced cyan and yellow fluorescent proteins, ECFP and EYFP, respectively. We replaced the ECFP-EYFP FRET pair with sequences encoding CyPet and YPet fluorescent proteins [16] and cloned the resultant *CyPet-RBP-YPet* sequence into the *pRSET* vector for bacterial expression driven by a *T7* promoter. Two *His_6_* sequences flanked the *CyPet-RBP-YPet* recombinant cassette to produce the “His_6_-CyPet-RBP-YPet- His_6_” (CRY) protein of approximately 87.6 kDa in mass.

**Figure 1:**
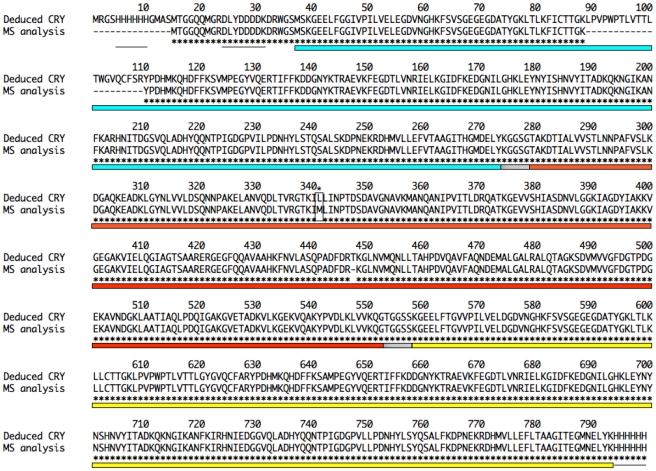
Mass Spectrometry for Amino Acid Sequence Verification of the Deduced CRY Protein. Protein samples were digested with gluC and trypsin proteases separately and analyzed using high resolution LC MS/MS. Mass spectrometry verified 97% of the deduced recombinant CRY sequence. The remaining 3% was either unidentified or represented 1 amino acid mismatch (*). Blue shading = CyPet sequence; Red shading = RBP sequence; Yellow shading = YPet sequence; Gray = linker sequences; Black Lines = His_6_ and Xpress™ tags.

The identity of the *E. coli*-expressed recombinant CRY protein and its FRET functionality were verified using LC MS/MS and ligand binding experiments, respectively. Mass spectrometry of the chimeric construct verified 97% of the amino acid sequence of the CRY fusion protein (Fig. 1). There was a leucine-to-methionine point mutation in the RBP portion of the sequence (L341M), which highlights the power of mass spectrometry in detecting small differences in deduced and analyzed amino acid sequences. Unidentified residues occurred in 21 positions within CyPet and a single position in the RBP portion of the chimeric protein. The precursor ion for the former was observed in several MS scans, however the MS/MS spectra were not of high enough quality to produce a positive identification with our strict filtering criteria. These discrepancies were not located in the ribose binding pocket or the hinge-twist functional domain of RBP [17] and were not expected to affect overall performance of the sensor cassette.

**Figure 2:**
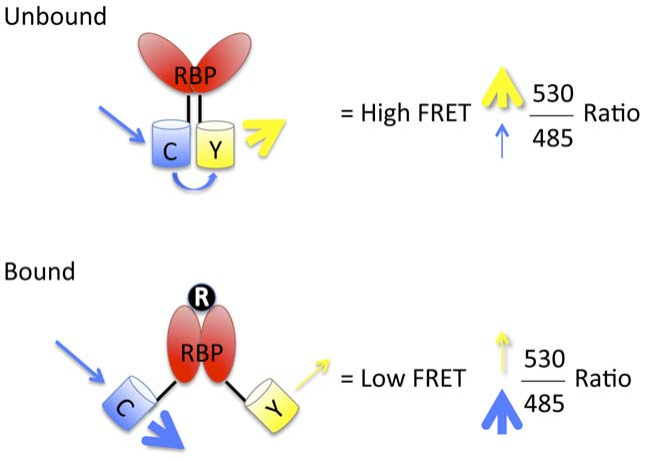
Model for Ribose-Induced Conformational Change in CRY and Associated Decrease in FRET. Unbound: In the absence of ribose, the ribose-binding protein (RBP) is in an open configuration, with C and Y, which are attached to the amino and carboxyl termini of RBP, respectively, in close proximity to each other. When CRY is excited at 435 nm, the emission from C excites Y, resulting in a high 530/485 emission intensity ratio. Bound: Binding of ribose (R) by RBP induces a conformational change in RBP that separates the amino and carboxyl termini of RBP, thereby increasing the distance between C and Y. This increase in the distance decreases the energy transfer between the two fluorescent proteins, thereby decreasing the 530/485 emission intensity ratio. Thus, increases in ribose concentrations result in decreases in FRET.

Prior to expression in the diatom, we verified the FRET functionality of *E. coli*-expressed recombinant CRY and Sil3-CRY by testing the sensitivity of the sensor constructs to added ribose. The binding of ribose to the receptor moves the amino and carboxyl termini of RBP further apart and increases the distance between CyPet and YPet, resulting in a reduction in FRET (Fig. 2). The binding of ribose to RBP would result in a decrease in the yellow/cyan (530/485 nm) relative fluorescence intensity ratio associated with the reduction in FRET.

**Figure 3:**
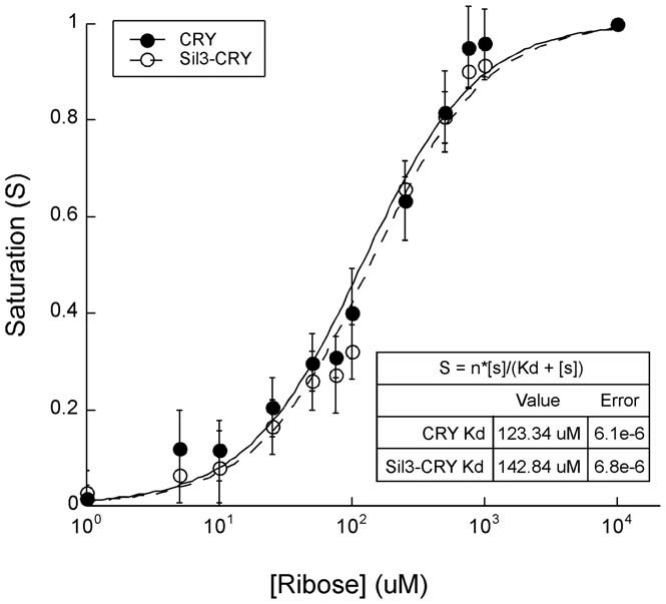
Ribose-Dependent FRET in Recombinant CRY and Sil3-CRY Expressed in *E. coli*. Isolated CRY and Sil3-CRY protein samples were treated with various concentrations of ribose ranging from 1 µM to 10 mM. The *K_d_*'s for CRY and Sil3-CRY are not significantly different from each other (see Materials and Methods for details on *K_d_* calculations and statistical analysis).

Consistent with this behavior, decreases in FRET efficiencies as a function of increasing ribose concentrations were evident from the saturation curves (Fig. 3). The respective dissociation constants (*K_d_*) of 123.3 µM for CRY and 142.8 µM for Sil3-CRY were not significantly different from each other or from the previously published *K_d_* of 119 µM for the ECFP-RBP-EYFP construct encoded by *FLIPrbs- F15A* [13] (p>0.1 for all comparisons). Thus, the substitution of CyPet and YPet for ECFP and EYFP, respectively, and addition of the Sil3 tag to CRY were not shown to significantly alter the receptor's affinity for ribose.

### CRY Expression in Diatoms

Having determined that the Sil3-CRY cassette functioned as expected *in vitro*, the diatom *T. pseudonana* was transformed with *pTpfcp/Sil3-CRY:fcp/nat*, the *Sil3-CRY* expression vector for the diatom, by high-pressure microparticle bombardment [11]. Both the *Sil3-CRY* expression cassette and the *nat* gene conferring nourseothricin resistance were encoded in this vector.

PCR amplification of genomic DNA isolated from transformed diatoms produced electrophoretic bands corresponding to both *CRY* and *nat* genes, which were absent in the untransformed wild-type diatoms. RT-PCR of first strand cDNA libraries resulted in bands corresponding to both the *CRY* and *nat* genes in transformed samples but not in the untransformed samples (data not shown). These results signified successful transformation and gene expression in the transformed diatoms.

**Figure 4:**
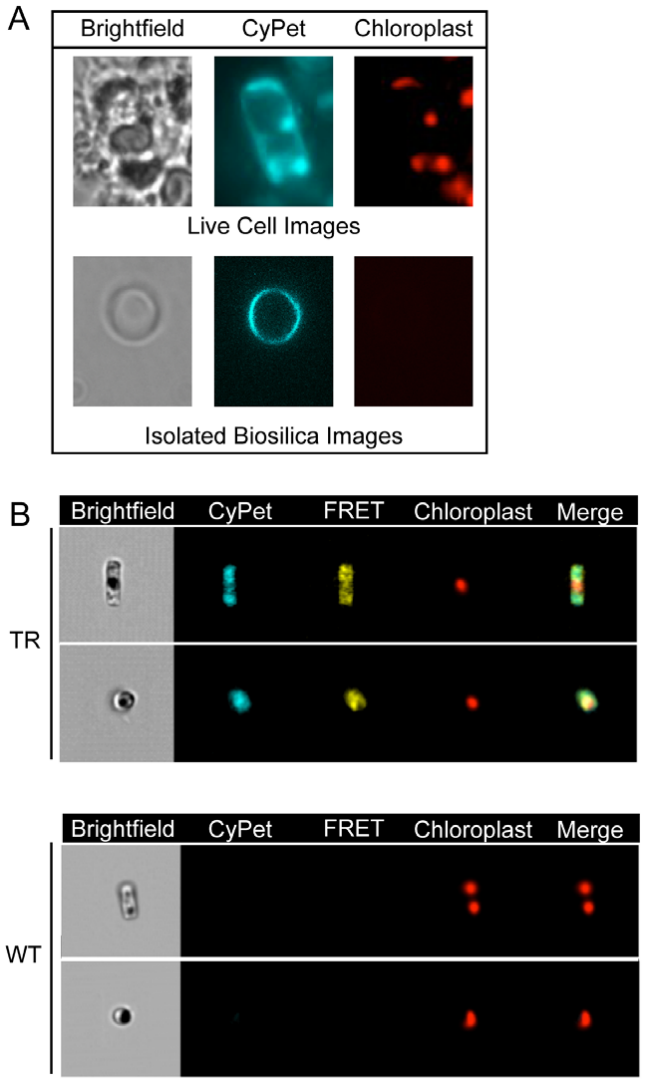
Fluorescent Images of Transformed and Untransformed Living Diatoms and Isolated Biosilica. (A) Fluorescence microscopy: Live transformed cells and isolated biosilica frustules were imaged for brightfield to display cell structure and for cyan fluorescence of CyPet and red auto-fluorescence to show their respective localization in the biosilica and chloroplasts. The lack of red auto-fluorescence in the isolated biosilica highlights the absence of chloroplasts and therefore, the lack of cellular material within the biosilica cell wall. (B) Imaging flow cytometry: *pTpfcp/Sil3-CRY:fcp/nat-* transformed (TR) and untransformed (WT) cells were imaged for brightfield, cyan fluorescence of CyPet, yellow fluorescence of YPet from FRET, and chloroplast auto-fluorescence. Cyan, yellow, and red images were merged to highlight the fluorescence of CyPet and YPet flanking the chloroplasts. Only the red auto-fluorescence of chloroplasts was detected in the WT cells.

We verified the presence of recombinant CRY in transformed diatoms and their isolated biosilica cell walls using fluorescence microscopy and imaging flow cytometry (Fig. 4A and 4B, respectively). We used fluorescence microscopy to image both transformed living cells and isolated frustules. This revealed a distinct perimeter of cyan fluorescence consistent with Sil3-CRY localization in the biosilica in both transformed cells and isolated biosilica shells (Fig. 4A). This fluorescence overlapped with the perimeter of the diatom in the brightfield images and was consistent with localization of CRY in the biosilica cell wall. An apparent co-localization of CRY in the chloroplast was attributed to bleed-through of the intense red auto-fluorescence of chlorophyll into the cyan and yellow channels. This observation was addressed by imaging flow cytometry.

The epifluorescence images captured by imaging flow cytometry were selected for further analysis using the following criteria: 1) only single cells in focus, not clumps, were imaged in the field of view, 2) images were positive for red chlorophyll auto-fluorescence, and 3) images were positive for FRET-derived yellow fluorescence. These criteria allowed us to view single living cells, signified by the presence of chlorophyll, and analyze the fluorescence intensity and patterning within the cell. The bleed-through of red chloroplast auto-fluorescence was compensated in the CyPet and YPet channels.

By gating each population as mentioned above, we analyzed 21.3% of the transformed population and 2.9% of the untransformed, wild-type population for further analysis. In the transformed subpopulation, the localization of the cyan and yellow fluorescence associated with CRY could be distinguished from that of the red auto-fluorescence of chlorophyll. Cells with cyan and yellow fluorescence adjacent to the red fluorescence of the chloroplast, rather than co-occurring in the same cellular location, were predominant (merged image in Fig. 4B TR). The intensity of cyan and yellow fluorescence in wild-type cells was usually faint or undetectable (Fig. 4B WT).

### FRET in Living Diatoms and Isolated Biosilica Cell Walls

**Figure 5:**
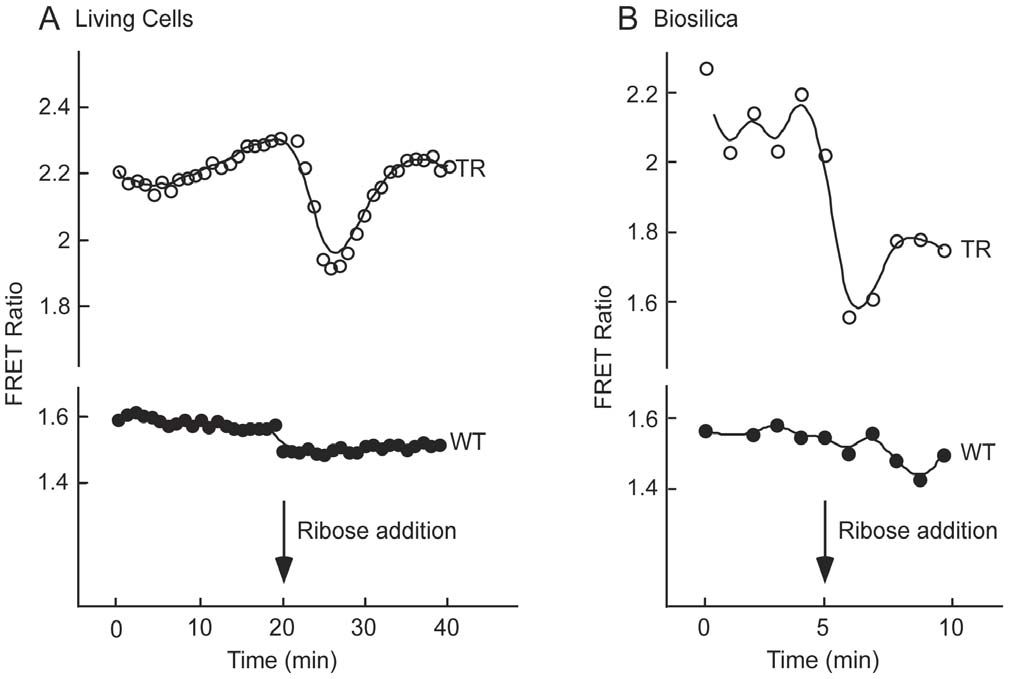
Ribose-Dependent FRET Imaging in Living, Transformed Diatoms and Isolated Biosilica. (A) Time lapse imaging of living cells was used to image the CyPet and YPet fluorescence before and after addition of 300 mM ribose. Fluorescent images were captured simultaneously in both fluorescent channels every min for 40 min. Ribose was added to the cells at 20 min following initial imaging. The FRET ratio (530/485 nm RFU ratio) decreases upon addition of ribose. (B) Similarly, time lapse imaging of biosilica cell walls was carried out for 10 min with the addition of 300 mM ribose after 5 min. The FRET ratio decreases in response to ribose addition.

**Figure 6:**
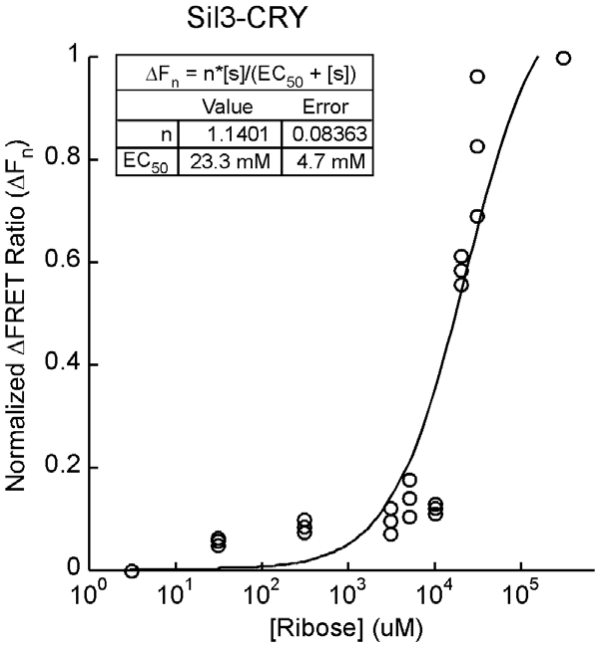
Concentration-Response of *pTpfcp/Sil3-CRY:fcp/nat-*transformed Diatoms Exposed to Ribose. Normalized ΔFRET values (ΔF_n_) for individual cells treated with ribose. The EC_50_ of 23.3±4.7 mM (mean ± S.E.) corresponds to the ribose concentration resulting in 50% of the maximal ΔF_n_ value.

Although initially uncertain whether conformational changes in RBP needed to drive changes in FRET would be constrained by the availability of substrate or steric hindrance related to immobilization of Sil3-CRY in the nanoporous architecture of the biosilica cell wall, ribose-induced changes in FRET were observed in both living transformed cells and isolated biosilica cell walls (response of representative cell shown in Fig. 5A and 5B). At a saturating level of 300 mM ribose, the FRET ratio (530/485 nm relative fluorescent units [RFU]) decreased by an average of 0.19±0.01 (1 S.E., n = 10) and 0.43±0.04 (1 S.E., n = 4) in transformed living cells and isolated biosilica cell walls, respectively. This difference between transformed cells and isolated biosilica was highly significant (p<0.001), as was the difference between these values and corresponding background changes in untransformed wild-type control structures (Fig. 5A and 5B) (p<0.05). A concentration-response curve for external ribose concentrations in living transformed diatoms indicated an EC_50_ of 23.3 mM (Fig. 6). Comparison of this value with the substrate affinity of the *E. coli* expressed CRY (Fig. 3) and the significantly greater reduction in FRET in the isolated biosilica when compared with the living cell suggested that the cell matrix hindered access of ribose to the silica-immobilized sensor.

## Discussion

The nanoscale, hierarchical organization of the diatom biosilica has been of interest as a model for materials synthesis, particularly in the context of silicification at mild pH and ambient temperature [18]. Initial research in this area focused on the use of polycationic peptides of diatoms, the silaffins [5], [19], in the chemical synthesis of hybrid silaffin-silica complexes from silicic acid. This work has been extended to the formation of immobilized enzyme reactors by adding enzymes such as catalase, horseradish peroxidase, and butyrylcholinesterase to the reaction and to the conjugation of polyamines to lysines to mimic the interaction between silaffins and polyamines in silica precipitation from silicic acid [20]–[22]. In both cases, the proteins are targeted for localization in the silica matrix.

The development of a transformation system for diatoms created the possibility of genetic manipulation of the biosilica structure through the expression of recombinant chimeric proteins that include silaffin or, more recently, cingulin as part of a chimeric fusion construct [23]. Both of the latter proteins can anchor the construct in the biosilica and have been tethered to functional proteins that localize in the biosilica and retain function when the biosilica is isolated from the cell [10], [12]. Proteins tested to date include those with multimeric subunit structures and cofactor requirements [12]. It was of interest to determine whether complex chimeric fusion proteins that incorporate multiple protein modules and have requirements for ligand-binding and large-scale motions could function within the spatial limitations of the diatom biosilica.

Because it was not known whether the Sil3 tag would hinder ribose binding by RBP, the conformational change of RBP in response to ribose, or the spatial interactions of CyPet and YPet, initial experiments examined FRET in purified *E. coli*-expressed recombinant CRY and Sil3-CRY. FRET for both CRY and Sil3-CRY functioned as expected, exhibiting *K_d_*'s similar to each other and to the *FLIPrbs- F15A*-encoded ECFP-RBP-EYFP construct [13]. We also demonstrated the sensor's functionality in living diatoms and isolated biosilica. Our study showed that a sensor cassette resulting from a fusion of four proteins with a biosilica-targeting tag was both localized to the diatom *T. pseudonana* cell wall and could undergo the necessary spatial rearrangements required for function as a FRET sensor.

Immobilization of functional recombinant proteins in diatom biosilica through *in vivo* bioassembly has now been demonstrated for GFP and HabB [10]; β-glucuronidase, glucose oxidase, galactose oxidase, and horseradish peroxidase [12]; and CRY (our current study). Their precise localization and density in the biosilica, which are of interest from an applied perspective related to the characteristics of hybrid silica materials, have yet to be reported. By inference from observations reported to date, they are likely to be bound to the external and internal surfaces of the biosilica and/or within the biosilica pores. The minimum radii for these proteins, estimated from molecular weights of 16,900 to 272,000 range from 1.69 to 4.27 nm [24] and are consistent with the apparent size requirements for inclusion in the pores of *T. pseudonana* (average diameter of valve pores of 18.2 nm [25]). Thus, barring other factors, several molecules of CRY (estimated molecular weight of 87,600 and minimum radius of 2.79 nm) could be accommodated within the architecture of a single pore structure. *T. pseudonana* with a valve diameter of 3 to 4 µm [25] is a relatively small diatom. Related species of *Thalassiosira*, such as *T. eccentrica*, *T. punctigera*, and *T. weissflogii* are larger and have considerably greater pore sizes [26], [27]. Moreover, the porosity of the frustule of *T. eccentrica* at 37% is sizeable [26]. These species appear to have the potential to accommodate higher amounts of the immobilized protein and could be candidates for genetic manipulations for biotechnological applications using approaches being developed for *T. pseudonana*.

With the advances currently being made in diatom engineering technologies and genetics, diatom-enabled functions such as catalysis, chelation, and biosensing emerge as potential applications, so long as the desired functionality can be encoded as proteins. Immobilizing proteins in mesoporous silica support matrices is advantageous for a wide variety of applications in medical diagnostics and therapy, biosensing, catalysis, and substrate interaction/inhibition. A better understanding of the design principles underlying the assembly of biosilica cell walls by diatoms should contribute to future *in vivo* bio-assembly of genetically-modified biosilica structures in transformed diatoms [10], [11] and inform bio-inspired chemical assembly of silica-immobilized protein structures *in vitro* [3], [4], [28]–[30].

## Materials and Methods

### Construction of Bacterial and Diatom Expression Construct

Standard cloning techniques were used to insert in frame the *CyPet, periplasmic ribose binding protein (RBP)*, and *YPet* genes into the multiple cloning site encoded in the *pRSET* bacterial expression vector. CyPet and YPet constitute a fluorescent protein FRET pair [16]. The *pRSET* vector contains a *T7* promoter driving expression of *CRY*. The *pRSET* vector is designed to encode two N-terminal tags, a polyhistidine (His_6_) tag for Ni-column purification and an Xpress™ epitope for detection with the Anti-Express™ antibody, and an enterokinase cleavage site upstream of the start site for CRY. We included an additional His_6_ tag on the C-terminal end of YPet for added nickel affinity. The fluorescent proteins were hinged to RBP through the insertion of a poly-linker sequence encoding Gly-Gly-Ser. Once sequence-verified, the *CyPet-RBP-YPet (CRY)* expression sequence was inserted downstream of the cDNA encoding Silaffin 3 *(Sil3)* isolated by RT-PCR from the diatom *T. pseudonana* (Strain CCMP 1335, Provasoli-Guillard National Center for Marine Algae and Microbiota, West Booth Harbor, MA). This resulted in two constructs for bacterial expression: *pRSET:CRY* and *pRSET:Sil3-CRY*. Conformational changes associated with binding of ribose to RBP are reported to result in an increased spatial separation of the fluorescent protein FRET pair and a reduction in FRET [13].

The genomic sequence of *Sil3* was used in the biosilica-targeting diatom expression vector *pTpfcp/Sil3nt* [10]. To create a single vector transformation system for expression of *CRY* targeted to the biosilica cell wall in diatoms, the *CRY* sequence was cloned into *pTpfcp/Sil3nt* between the genomic *Sil3* and *fcp* terminator sequences. The antibiotic selection gene *nat* was inserted downstream of a second *fcp* promoter conferring antibiotic resistance to nourseothricin, thus resulting in the diatom expression vector *pTpfcp/Sil3-CRY:fcp/nat*.

The *CyPet* and *YPet* vectors were provided by Patrick Daugherty, the University of California, Santa Barbara; the *RBP* sequence was cloned from *FLIPrbs- F15A* provided by Wolff Frommer, Carnegie Institution for Science, Stanford University; and *pTpfcp/Sil3nt* and *pTpfcp/nat* were provided by Nicole Poulsen and Nils Kröger, Georgia Institute of Technology.

### Expression and Isolation of *E. coli*-Expressed Recombinant Proteins

TurboCells BL21 (DE3) competent *E. coli* (Genlantis, Inc., San Diego, CA) transformed with the bacterial expression constructs were grown overnight at 37° C. OD_600_ measurements were taken and cultures were diluted back to an OD_600_ of 0.5. Cells were grown at 37° C until the OD_600_ reached 1.0. Protein expression was then induced by the addition of IPTG (isopropyl B-D-thiogalactoside) at a concentration of 100 µM; cultures were transferred to 26° C and grown overnight in the dark. Expressed protein was isolated using Ni-affinity chromatography (His-Bind resin, Novagen, Inc.). Cells were lysed by sonication, centrifuged, and the lysate was filter-sterilized before being applied to the column. Protein-bound columns were washed with increasing concentrations of imidazole ranging from 5 mM to 500 mM. Selective washes were pooled and concentrated in a 50-kDa Amicon filtration system (EMD MilliPore). Protein purification was monitored by SDS-PAGE using a 10% polyacrylamide gel (Bio-Rad Laboratories, Inc.).

### Mass Spectrometry of *E. coli*-Expressed Recombinant Protein

CRY protein samples isolated as described above were buffer-exchanged into 200 mM ammonium bicarbonate, digested with gluC and trypsin proteases separately, and analyzed by high resolution LC MS/MS on the LTQ Velos Orbitrap (Thermo Scientific, Waltham, MA) platforms. Initial peptide identifications were made by SEQUEST using an *E. coli* database containing the CRY protein sequence. The leucine-to-methionine point mutation, L341M, was identified using in-house *de novo* sequencing tools [31] and could also be identified by SEQUEST when using the CRY sequence including the point mutation. The other peptide sequences identified by SEQUEST were also verified by the in-house *de novo* sequencing tools.

### *In vitro* Analysis of FRET in *E. coli*-Expressed Recombinant Proteins

Substrate titration curves for purified recombinant proteins were performed on a Synergy-HT spectrofluorimeter with the excitation wavelength set to 430 nm (CyPet excitation) and the emissions set to 485 nm (CyPet emission) and 530 nm (YPet emission). Isolated protein samples were diluted into 20 mM sodium phosphate buffer pH 7.0, and FRET was determined as the 530/485 nm emission intensity ratio.

The dissociation constant (*K_d_*) for each protein was determined by plotting saturation (S) against the ribose concentration [s] over the range 1 µM to 10 mM, using a method modified from Lager et al [13]. Saturation was calculated as
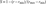
where r is the 530/485 nm fluorescence ratio for a given ribose concentration, r_min_ is the 530/485 ratio at saturation, and r_max_ is the 530/485 ratio with no ribose addition. An increase in S corresponds to a decrease in the FRET efficiency. The *K_d_* of each receptor was estimated from the following relationship:
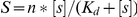
where n is the number of binding sites, and [s] is ribose concentration.

Statistics were performed using Prism v4 GraphPad software, 2003. Data sets were compared to each other and published values using non-linear regression with the number of binding sites constrained to n = 1. An F-test was used to test for a common *K_d_* parameter for CRY and Sil3-CRY. The *K_d_* of each was also tested against that of ECFP-RBP-EYFP encoded by *FLIPrbs- F15A* [13].

*Diatom Culture and Transformation. T. pseudonana* (Strain CCMP 1335) was grown in f/2 media (Provasoli-Guillard National Center for Marine Algae and Microbiota, West Booth Harbor, MA) supplied with aeration and constant light at 18–20°C. Regular sparging of the cultures with CO_2_ kept the pH between 8 and 8.5, and cultures were supplemented with 100 µg/ml penicillin and streptomycin weekly.

Diatom transformation by microparticle bombardment using the PDS-1000/He particle delivery system (Bio-Rad, Hercules, CA) was performed as described previously [11]. Nourseothricin-resistant cells were used for analysis of fluorescence localization and FRET response.

### Gene Expression Analysis

Isolation of genomic DNA from *T. pseudonana* was performed using a modified version of the Genomic DNA Isolation Kit protocol (Promega, Madison, WI). Approximately 2×10^8^ diatom cells were harvested by centrifugation and resuspended in 600 µl Nuclei Lysis Solution. Cells were lysed by bead beating followed by a 65° C incubation for 15 min. Cells were treated with RNase A and protein was precipitated using the Protein Precipitation Solution. DNA was isolated by isopropanol precipitation followed by phenol∶chloroform extraction to remove residual proteins. DNA samples were resuspended in TE. The success of transformation was determined by PCR for the *CRY* and *nat* inserts followed by agarose gel electrophoresis.

Total RNA extraction from *T. pseudonana* was performed using a modified version of the RNeasy Plant Kit (QIAGEN, Valencia, CA). Samples were lysed in RLT buffer by bead beating for 3 min followed by ethanol precipitation and column purification per manufacture's protocol. RT-PCR using oligo dT primers (Invitrogen, Carlsbad, CA) was performed per the manufacture's protocol for the SSIII reverse transcriptase (Invitrogen, Carlsbad, CA) using 1 µg of RNA. Analysis of gene expression was determined by PCR and agarose gel electrophoresis.

### Isolation of Frustules

Frustules were isolated using the procedure described by Poulsen et al. 2007 [10], modified to achieve satisfactory FRET analysis. Detergents and EDTA were omitted from the buffer. Cells were sonicated using five sequential pulses of 30 sec (Fisher Scientific Model 500 Sonic Dismembrator, amplitude of 10%) and allowed to settle in a microcentrifuge rotating at only 0.8 rpm. Frustules collected in this manner were then imaged and analyzed for FRET as described below for live cells.

### Fluorescence Localization Imaging

Cellular fluorescence was captured using an ImageStream^X^® high-resolution imaging flow cytometer (Amnis, Co., Seattle, WA). Approximately 10,000 cell samples were excited using a 405 nm laser, and five images per cell were collected in the cyan, yellow, red, brightfield and side scatter channels. A 66SP filter was inserted into the laser line to minimize the chloroplast auto-fluorescence. Cyan and yellow fluorescent images were compensated for red fluorescence bleed-through. Following the flow cytometry image capture, samples were gated to produce populations of captured single-cell images that were in focus and displayed the stereotypical red auto-fluorescence indicative of living cells. Brightness and contrast for the cyan and yellow fluorescence intensities were adjusted similarly for each channel in TR and WT samples.

Biosilica-localized fluorescence was imaged on a DM IRB inverted light microscope (Leica, Inc.) using a SPOT camera and imaging analysis software (SPOT, Inc.). Living cells and biosilica cell walls collected from cell cultures were imaged for cyan and red fluorescence. Brightness and contrast were adjusted accordingly.

### FRET Imaging and Analysis

Cells were examined using a DM IRB inverted light microscope (Leica, Inc.), and images were captured with a Photometrics CoolSNAP EZ camera attached to a DV2 image splitter with cyan and yellow emission filters. Cell images were post-processed by pseudo-coloring each image appropriately using MetaMorph® Software and overlapping images for protein localization using Photoshop software (Adobe, Inc.). Brightness and contrast were adjusted similarly for each image.

For FRET analysis, cells or frustules were immobilized on collagen-coated 35 mm petri dishes (MatTek, Ashland, MA), photobleached for five minutes at 430 nm to minimize the bleed-through of the red auto-fluorescence of chloroplasts into the CyPet and YPet channels, then excited at 430 nm by pulsing at one minute intervals up to 40 min. Images for 485 nm and 530 nm emission spectra were processed using MetaMorph® Software. FRET was measured as the ratio of the RFU at 530 nm and 485 nm, corrected for background fluorescence of each wavelength at a neutral location on the plate. FRET was calculated on a pixel-by-pixel basis, and FRET curves were constructed by plotting the 530/485 nm ratio as a function of time.

To construct the concentration-response curve for diatoms exposed to ribose, ΔF was first normalized to ΔF_max_, following correction of each with ΔF_min_, to provide ΔF_n_:
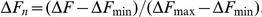



The EC_50_ for cellular FRET (the concentration at which 50% of the maximal ΔF_n_ is observed) was determined by plotting ΔF_n_ as a function of the ribose concentration [s]:
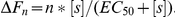



## References

[1] Chen J, Doumamidis H, Lyons K, Murday J, Roco MC (2007). Manufacturing at the Nanoscale.. National Nanotechnology Initiative Workshop Report.

[2] Gordon R, Parkinson J (2005). Potential roles for diatomists in nanotechnology.. Journal of Nanoscience and Nanotechnology.

[3] Chang CH, Wang W, Gutu T, Gale DK, Jiao J (2009). Self-Assembly of Nanostructured Diatom Microshells into Patterned Arrays Assisted by Polyelectrolyte Multilayer Deposition and Inkjet Printing.. Journal of the American Chemical Society.

[4] Kent MS, Murton JK, Satija S, Kuzmenko I, Simmons BA (2009). Nanosilica Formation at Lipid Membranes Induced by Silaffin Peptides.. Structure-Property Relationships in Biomineralized and Biomimetic Composites.

[5] Kröger N, Deutzmann R, Sumper M (1999). Polycationic peptides from diatom biosilica that direct silica nanosphere formation.. Science.

[6] Kröger N, Sandhage KH (2010). From Diatom Biomolecules to Bioinspired Syntheses of Silica- and Titania-Based Materials.. Mrs Bulletin.

[7] Sandhage KH, Bao ZH, Weatherspoon MR, Shian S, Cai Y (2007). Chemical reduction of three-dimensional silica micro-assemblies into microporous silicon replicas.. Nature.

[8] Rorrer GL, Jeffryes C, Gutu T, Jiao J (2008). Metabolic Insertion of Nanostructured TiO_2_ into the Patterned Biosilica of the Diatom *Pinnularia* sp by a Two-Stage Bioreactor Cultivation Process.. Acs Nano.

[9] Rorrer GL, Jeffryes C, Gutu T, Jiao J (2008). Two-stage photobioreactor process for the metabolic insertion of nanostructured germanium into the silica microstructure of the diatom *Pinnularia* sp.. Materials Science & Engineering C-Biomimetic and Supramolecular Systems.

[10] Poulsen N, Berne C, Spain J, Kröger N (2007). Silica immobilization of an enzyme through genetic engineering of the diatom *Thalassiosira pseudonana*.. Angewandte Chemie-International Edition.

[11] Poulsen N, Chesley PM, Kröger N (2006). Molecular genetic manipulation of the diatom *Thalassiosira pseudonana* (Bacillariophyceae).. Journal of Phycology.

[12] Sheppard V, Scheffel A, Poulsen N, Kröger N (2012). Live diatom silica immobilization of multimeric and redox-active enzymes.. Appl Environ Microbiol.

[13] Lager I, Fehr M, Frommer WB, Lalonde SW (2003). Development of a fluorescent nanosensor for ribose.. Febs Letters.

[14] Campbell RE, Carlson HJ (2009). Genetically encoded FRET-based biosensors for multiparameter fluorescence imaging.. Current Opinion in Biotechnology.

[15] Newman RH, Fosbrink MD, Zhang J (2011). Genetically Encodable Fluorescent Biosensors for Tracking Signaling Dynamics in Living Cells.. Chemical Reviews.

[16] Nguyen AW, Daugherty PS (2005). Evolutionary optimization of fluorescent proteins for intracellular FRET.. Nature Biotechnology.

[17] Mowbray SL, Cole LB (1992). 1.7 Angstrom X-Ray Structure of the Periplasmic Ribose Receptor from *Escherichia coli*.. Journal of Molecular Biology.

[18] Patwardhan SV (2011). Biomimetic and bioinspired silica: recent developments and applications.. Chemical Communications.

[19] Kröger N, Poulsen N (2008). Diatoms-From Cell Wall Biogenesis to Nanotechnology.. Annual Review of Genetics.

[20] Luckarift HR, Johnson GR, Spain JC (2006). Silica-immobilized enzyme reactors; application to cholinesterase-inhibition studies.. Journal of Chromatography B-Analytical Technologies in the Biomedical and Life Sciences.

[21] Naik RR, Tomczak MM, Luckarift HR, Spain JC, Stone MO (2004). Entrapment of enzymes and nanoparticles using biomimetically synthesized silica.. Chemical Communications.

[22] Wieneke R, Bernecker A, Riedel R, Sumper M, Steinem C (2011). Silica precipitation with synthetic silaffin peptides.. Organic & Biomolecular Chemistry.

[23] Scheffel A, Poulsen N, Shian S, Kröger N (2011). Nanopatterned protein microrings from a diatom that direct silica morphogenesis.. Proceedings of the National Academy of Sciences of the United States of America.

[24] Erickson HP (2009). Size and Shape of Protein Molecules at the Nanometer Level Determined by Sedimentation, Gel Filtration, and Electron Microscopy.. Biological Procedures Online.

[25] Hildebrand M, York E, Kelz JI, Davis AK, Frigeri LG (2006). Nanoscale control of silica morphology and three-dimensional structure during diatom cell wall formation.. Journal of Materials Research.

[26] Losic D, Rosengarten G, Mitchell JG, Voelcker NH (2006). Pore architecture of diatom frustules: Potential nanostructured membranes for molecular and particle separations.. Journal of Nanoscience and Nanotechnology.

[27] Vrieling EG, Beelen TPM, van Santen RA, Gieskes WWC (2000). Nanoscale uniformity of pore architecture in diatomaceous silica: A combined small and wide angle X-ray scattering study.. Journal of Phycology.

[28] Li L, Jiang ZY, Wu H, Feng YN, Li J (2009). Protamine-induced biosilica as efficient enzyme immobilization carrier with high loading and improved stability.. Materials Science & Engineering C-Materials for Biological Applications.

[29] Marner WD, Shaikh AS, Muller SJ, Keasling JD (2009). Enzyme Immobilization via Silaffin-Mediated Autoencapsulation in a Biosilica Support.. Biotechnology Progress.

[30] Spain JC, Luckarift HR, Naik RR, Stone MO (2004). Enzyme immobilization in a biomimetic silica support.. Nature Biotechnology.

[31] Shen YF, Tolic N, Hixson KK, Purvine SO, Anderson GA (2008). De novo sequencing of unique sequence tags for discovery of post-translational modifications of proteins.. Analytical Chemistry.

